# Device-Free Human Activity Recognition with Low-Resolution Infrared Array Sensor Using Long Short-Term Memory Neural Network

**DOI:** 10.3390/s21103551

**Published:** 2021-05-20

**Authors:** Cunyi Yin, Jing Chen, Xiren Miao, Hao Jiang, Deying Chen

**Affiliations:** College of Electrical Engineering and Automation, Fuzhou University, Fuzhou 350108, China; 200110004@fzu.edu.cn (C.Y.); mxr@fzu.edu.cn (X.M.); jiangh@fzu.edu.cn (H.J.); 200120067@fzu.edu.cn (D.C.)

**Keywords:** human activity recognition (HAR), low-resolution infrared array sensor, long short-term memory (LSTM)

## Abstract

Sensor-based human activity recognition (HAR) has attracted enormous interests due to its wide applications in the Internet of Things (IoT), smart homes and healthcare. In this paper, a low-resolution infrared array sensor-based HAR approach is proposed using the deep learning framework. The device-free sensing system leverages the infrared array sensor of 8×8 pixels to collect the infrared signals, which can ensure users’ privacy and effectively reduce the deployment cost of the network. To reduce the influence of temperature variations, a combination of the J-filter noise reduction method and the Butterworth filter is performed to preprocess the infrared signals. Long short-term memory (LSTM), a representative recurrent neural network, is utilized to automatically extract characteristics from the infrared signal and build the recognition model. In addition, the real-time HAR interface is designed by embedding the LSTM model. Experimental results show that the typical daily activities can be classified with the recognition accuracy of 98.287%. The proposed approach yields a better result compared to the existing machine learning methods, and it provides a low-cost yet promising solution for privacy-preserving scenarios.

## 1. Introduction

Human activity information plays a crucial role in human health monitoring and life enhancement in intelligent buildings. The advent of the Internet of Things (IoT) era has integrated human activity recognition (HAR) with intelligent life, bringing great changes to human life [1]. For example, home automation tasks, e.g., intelligent control of the lighting and air conditioning, can be achieved by HAR. Moreover, it can be implemented in monitoring the health of the elderly to obtain their health condition indirectly.

The conventional approach to detect human activities mainly relies on wearable devices [2], where users monitor their activity by wearing a wearable device on their bodies. For example, accelerometers have been applied to joints and torso positions to recognize the movements of each part [3,4,5]. Inertial sensors and gyroscopes have been incorporated into movable devices for HAR [6]. Igor B et al. [7] proposed a method based on an accelerometer combined with the SVM algorithm to identify common behaviors of humans. Gui H et al. [8] proposed a wearable device using RFID combined with a three-dimensional accelerometer to realize the recognition of human activities. Xue Q et al. [9] proposed a hidden Markov model (HMM)-based data analysis approach, where the approach learns from raw RFID data and applies this to analyze the data. HAR based on RFID is also a wearable device approach. The difficulty for the portability and convenience of wearable devices to meet the requirements of most use scenarios is readily apparent. The camera-based approach has been applied to HAR with artificial intelligence, and image recognition has made enormous strides [10]. Images were collected automatically by camera for HAR to liberate humans from wearable devices [11]. Ni et al. [12] comprehensively evaluated these extended feature representation methods based on the combination of color cameras and depth sensors and applied them to HAR by combining the color and depth information of human actions. Sanal K et al. [13] used the genetic algorithm and random forest (RF) for feature classification such as directed gradient histograms (HOGs), color and GIST of HAR. The camera-based approach relies on the light conditions. It is seriously affected by poor light conditions or occlusion. In addition, it is easy for the camera to invade humans’ privacy, and it is destined to be restricted in places with higher privacy, such as bedrooms and toilets [14]. It is obviously impractical to adopt the camera-based approach in the privacy-sensitive environment of certain rooms. The WiFi signal for HAR research has attracted more attention because of the popularity of routers and wireless network coverage. There have been advancements in WiFi research. Kun Z et al. [15] proposed WiFi-based channel state information (CSI) on an HAR system. The characteristics of the different activities were extracted through unsupervised machine learning methods. This approach lowered labor costs whilst efficiently identifying different human activities. Schfer J et al. [16] conducted fine-grained detection of HAR based on CSI and combined machine learning and deep learning networks to recognize human behavior in line-of-sight scenes and non-line-of-sight scenes in indoor environments. Wei W et al. [17] proposed a CSI activity model based on HMM that quantifies the relation between human movement speeds and human activities. Most CSI-based methods need the deployment of multiple expensive sensors for networking.

Infrared sensors have attracted more and more attention from HAR researchers for the advantages such as convenient deployment, low cost and privacy protection. LIL G et al. [18] employed different heats detected by infrared signals for HAR research. Chen et al. [19] put infrared sensors on the ceiling and walls to capture the three-dimensional image information of humans and detect their fall behavior. Yun J et al. [20] detected the human movement direction based on analog pyroelectric infrared (PIR) sensor signals. However, more complex HAR is difficult to achieve because PIR is limited by the number of infrared signals. Cai L et al. [21] utilized three PIR sensors in developing an infrared sensing system which aims to handle the difficulties caused by occlusion and a limited coverage area. Qiu G et al. [22] used the Gaussian mixture hidden Markov model (GS-HMM) to process the infrared signals of four PIR sensors for HAR. An infrared array sensor that can provide more infrared signals than one PIR was proposed [23]. The increase in signal dimensions deems it possible for the sensor to be used in HAR. Munk. G et al. [24] proposed a device-free unobtrusive indoor human posture recognition system leveraging the infrared sensor of 48 × 16 pixels and a deep convolutional neural network (DCNN). Chi S et al. [25] obtained thermal images from high-resolution infrared sensors of 120 × 160 and fed them into the super-resolution convolutional neural network (SRCNN) model to recognize the different activities. Md U et al. [26] proposed a thermal image-based HAR approach using features and a deep neural network. Bat G et al. [27] proposed an HAR method based on thermal images, which uses a convolutional neural network (CNN) to identify human activities without a light source. The deployment of lower-resolution infrared sensors makes it possible not only to reduce costs but also to protect the privacy of users to a large extent. The infrared data collected by lower-resolution sensors have lower dimensions, and it is more difficult to extract the infrared signal features. It is a great challenge for lower-resolution infrared array sensors to achieve similar accuracy to higher-resolution infrared array sensors.

In this paper, a deep learning-based HAR approach is developed using a low-cost and low-resolution infrared array sensor. The signals of 8×8 pixel thermal images from the infrared sensor are used to identify the human activities. The low-resolution infrared sensor can maintain the users’ privacy and considerably reduce the hardware costs. However, it is challenging to extract the characteristics of different activities from the limited low-dimensional infrared signal. To address this issue, the 8×8 pixel thermal images are converted to a temporal sequence for training. A long short-term memory (LSTM) neural network, a powerful variation of the recurrent neural network (RNN) architecture, is utilized to learn the representative features from the sequential infrared signals and build the classification model. LSTM has great advantages in dealing with time series data. The infrared signal used for training is composed of time series data. The input of LSTM is composed of three parts, namely, the input at the current moment, the output at the previous moment and the long-term state at the previous moment which saves the long-term information. In particular, the long-term state retains information from the previous moment.

In addition, LSTM has three special gate structures: a forget gate, an input gate and an output gate. Therefore, the network has the function of long-term memory, which improves the relevance of time series data. To reduce the influence of temperature variations, a noise reduction method called J-filter is combined with the Butterworth filter to handle the original infrared signals. In addition, a real-time HAR interface equipped with the LSTM model is designed to realize high-accuracy recognition processes.

The main contributions of this paper are as follows:

1. A low-resolution infrared array sensor of 8 × 8 pixels is applied. It is a low-cost, privacy-protective and device-free sensor that establishes a good trade-off between performance and cost.
2. A noise reduction method called J-filter is proposed to address the heterogeneity issue of the background temperature for infrared signal preprocessing. The original absolute infrared signals are replaced by the relative values extracted by J-filter to eliminate the influence of the temperature and improve the robustness for HAR.
3. A deep learning model based on LSTM is used to extract infrared signal features. The features of low-dimensional infrared signals can be depth mined in the model training process. The well-trained LSTM model can quickly and accurately recognize the human activities through the designed real-time interface.

The rest of this paper is organized as follows. In Section 2, we describe the proposed framework of HAR. The experimental results and analysis are presented in Section 3. Section 4 draws conclusions from the results and discusses our future work plan.

## 2. System Framework Overview and Methodology

### 2.1. System Framework Overview

**Figure 1:**
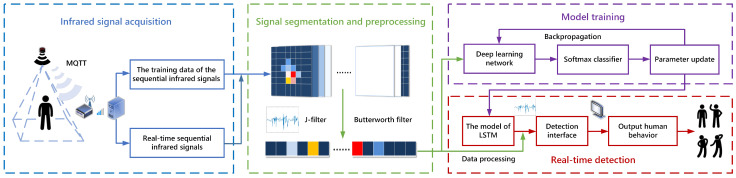
Schematic diagram of HAR method based on deep learning.

An HAR method based on deep learning is proposed. A low-resolution infrared array sensor of 8 × 8 pixels is leveraged in the HAR approach. The system is divided into four steps, as shown in Figure 1: infrared signal acquisition, signal segmentation and preprocessing, model training and real-time detection. The infrared signals of different activities are collected by the infrared array sensor installed on the ceiling of the room. With the Message Queuing Telemetry Transport (MQTT) protocol, the data receiver obtains data by subscribing to related topics directly. After collecting the data, the data stream is segmented according to a specific number of frames, and the signal samples corresponding to different activities are represented by continuous multi-frame data. After the segmentation, the data are preprocessed, and the J-filter eliminates the background noise caused by the temperature variation. The data are converted into a standard data format and then enter the feature extraction process. The infrared signal is trained through the constructed model based on deep learning, and the effect of the model is tested. After training the model, the infrared signal collected in real time is input to the real-time detection module for judgment. Then, the information of human activities is output.

### 2.2. Infrared Signal Acquisition

**Figure 2:**
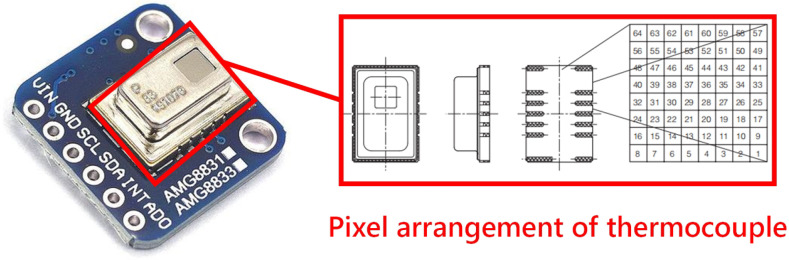
The 8×8 pixel low-resolution infrared array sensor of the Grid-EYE AMG8833 module.

The Grid-EYE AMG8833, a low-resolution infrared array sensor of 8×8 pixels, made by Panasonic is used in this study. As shown in Figure 2, the sensor contains 64 thermocouples with 8 vertical rows and 8 horizontal rows. The output temperature of the thermocouples ranges from −20 to $80{\;}^{\circ }C$, and the temperature accuracy is $\pm 2.5{\;}^{\circ }C$. It has a detection range of 7 m [28]. It is suitable for HAR because of its low price, ease of installation and steady performance. The synchronous serial bus $I^{2}C$ can be supported by the sensor, and the temperature value obtained by the sensor is returned to the connected microprocessor through $I^{2}C$. The microprocessor chooses ESP8266, as it has the advantages of low power consumption and stable performance [29]. The processor supports the standard IEEE 802.11 b/g/n protocol, and NodeMcu can be developed through the WiFi serial port communication module.

The infrared signals of different activities are collected by the low-resolution infrared array sensor. Then, the signals are sent to ESP8266. The sensor collects signals at a fixed frequency to generate sequential infrared signals. The sequential infrared signal is published and transmitted through the WiFi serial port communication module of the microprocessor and the MQTT protocol. The sequential infrared signal corresponding to different activities of humans is obtained after the data receiver subscribes to the topic. A server acts as a data receptor, and the sequential infrared signal is stored in the server. Data analysis and processing are performed concurrently on this server.

### 2.3. Signal Segmentation and Preprocessing

#### 2.3.1. Segmentation

**Figure 3:**
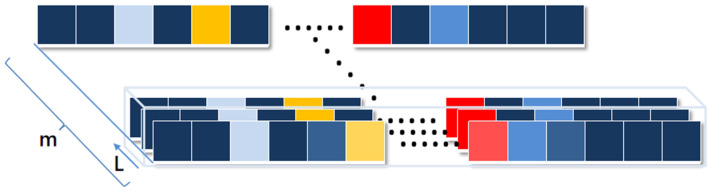
Segmentation of the sequential infrared signals.

Human activity occurs in a time frame. A single infrared signal frame cannot completely reflect human activity changes, particularly for dynamic activity. Therefore, a period of infrared signals (hereinafter referred to as sequential infrared signals) is used to reflect the human activity. Infrared signals are collected for a period of time for each activity to ensure data continuity. It becomes a necessary step to segment the sequential infrared signals. Human activity is not shown for small-cut length data, while if the length of each sample is too large, the cost of training increases. It is challenging to cut the sequential infrared signals into suitable size samples. As shown in Figure 3, the sequential infrared signals of *m* frames are divided into a sliding window of length *L*. In other words, a sequential infrared signal with *m* frames is segmented. The segmented sequential signal generates a sample with *L* frames. Eventually, there are m/L samples that can be generated in total by the sequential signal with *m* frames.

#### 2.3.2. Signal Preprocessing

**Figure 4:**
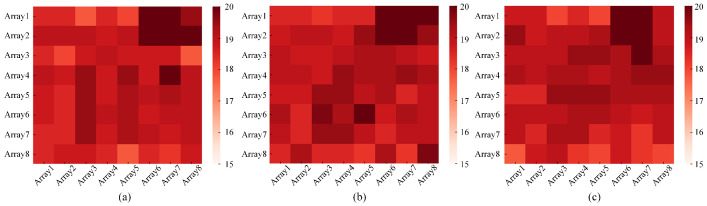
The heatmaps of the original infrared signals corresponding to standing at different temperatures: (**a**) at $14{\;}^{\circ }C$; (**b**) at $17{\;}^{\circ }C$; (**c**) at $20{\;}^{\circ }C$.

**Figure 5:**
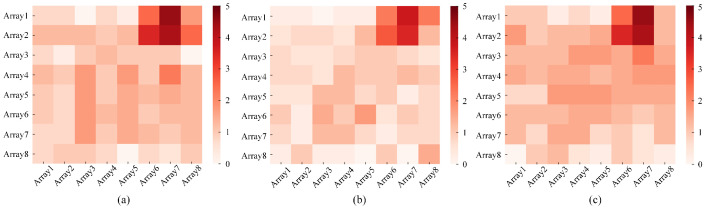
The heatmaps of the infrared signals processed by the J-filter method for standing at different temperatures: (**a**) at $14{\;}^{\circ }C$; (**b**) at $17{\;}^{\circ }C$; (**c**) at $20{\;}^{\circ }C$.

The infrared signal is interfered with by many factors during the collection process: for example, the influence of temperature changes and thermal dissipation of electrical equipment. It is essential to filter and reduce the noise from the collected infrared signals. Figure 4 shows the heatmaps of infrared signals corresponding to the standing activity collected by the sensor at $14{\;}^{\circ }C$, $17{\;}^{\circ }C$ and $20{\;}^{\circ }C$. Through observation, it can be found that a larger disturbance appears in the heatmaps when the room temperature changes. It can be seen that the temperature change has a great influence on the infrared signal under the same activity, leading to the difficulty in extracting features under different activities. Reducing the effect of temperature changes on infrared signals is an absolute priority. A more realistic noise reduction method is proposed specifically for infrared signals, called J-filter. After the infrared signal of a frame is input to the J-filter, the elements of the frame are sorted by size first, and the smallest value $T_{min}$ is selected. $T_{min}$ is the lowest temperature signal detected by the sensor at this moment, and the current indoor temperature can be indirectly reflected by $T_{min}$. Then, the relative change value $T_{n}^{\prime }$ of each element in the frame at the current temperature is obtained by subtracting $T_{min}$ from the element $T_{n}$ in the frame. The element at the original position in the frame is covered by $T_{n}^{\prime }$. Finally, the updated infrared signal of the frame is used as the output after noise reduction. Figure 5 corresponds to the original infrared signals of Figure 4 and heatmaps of the infrared signal after noise reduction by the J-filter. Through observation and comparison, it can be found that the infrared signals corresponding to the same activity at different temperatures after J-filter processing have a more obvious characteristic distribution. Under the application of the J-filter, the influence of temperature changes is eliminated to a large extent, which is more beneficial to the data feature extraction in subsequent model training. The effect of the J-filter will be demonstrated in subsequent chapters.

**Figure 6:**
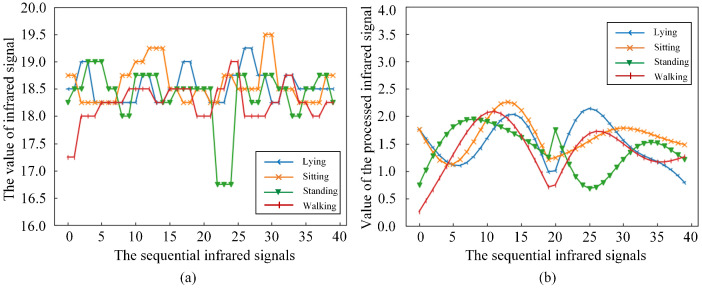
The infrared signal comparison chart before and after data processing. (**a**) The original infrared signal. (**b**) The infrared signal processed by J-filter and Butterworth filter.

The fluctuation of the sensor itself and the random noise generated in the environment can easily cause signal mutation. This signal mutation is not caused by changes in human activity or location. The Butterworth filter can reduce fluctuations to achieve smooth data [30]. For an abrupt infrared signal, the Butterworth filter is used to reduce its influence on the real signal. The effect of the Butterworth filter on the infrared signal is shown in Figure 6. Figure 6a is the original sequential infrared signal corresponding to the activities of lying, sitting, standing and walking. Figure 6b is the infrared signal processed by the J-filter and the Butterworth filter. By comparison, it can be easily found that the infrared signals corresponding to various activities have more obvious differences after being processed by the J-filter and the Butterworth filter. Due to the complexity of human activity and the influence of factors such as the changes in human location, it is impossible to accurately achieve HAR through the similarity matching of infrared signals. The deep learning method is used to extract the features of the data automatically and train the model to obtain the depth-sensing HAR model of the sequential infrared signals.

### 2.4. Model Training

**Figure 7:**
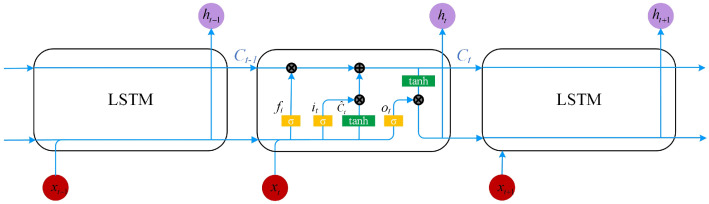
The structure of LSTM.

LSTM has the ability to process long-term information [31]. It solves the problem of gradient disappearance in recurrent neural networks (RNN) [32]. In particular, it has achieved great success in processing time series data [33]. LSTM is selected to train time series infrared signals. The structure of LSTM is shown in Figure 7.

The main structure of the network is the rectangular part in the middle. There are three inputs in LSTM totally, which are the input value $x_{t}$ at time t, the output value $h_{t-1}$ at the previous time and the long-term state $C_{t}$ storing long-term information at the previous time. Compared with an RNN, there are three more control structures in LSTM: a forget gate, an input gate and an output gate, in order to realize the control of the long-term state $C_{t}$. It is thanks to these special structures that LSTM has control over the long-term state $C_{t}$. The forget gate controls the long-term state $C_{t-1}$ at the previous moment, and it controls how many long-term states $C_{t-1}$ at the previous moment are retained for the long-term state $C_{t}$ at the current moment by calculating the probability, which can be expressed as

(1) $$f_{t}=\sigma \left(W_{f}\cdot \left[h_{t-1},x_{t}\right]+b_{f}\right)$$

where $f_{t}$ is the output of the forget gate, σ represents the use of sigmoid as the activation function in the forget gate, $W_{f}$ is the weight matrix and $b_{f}$ is the bias term of the forget gate. The input gate mainly controls the input at the current moment. How many inputs will be saved to the current long-term state $C_{t}$ is also controlled by calculating the probability. The input gate consists of two parts: they are sigmoid activation and tanh activation. The first part uses the sigmoid function to process the data and then outputs them. The second part uses the tanh function to process the data and obtain the output ${\tilde{C}}_{t}$. The corresponding processing formula is as follows:

(2) $$\left\{\begin{array}{c} i_{t}=\sigma \left(W_{i}\cdot \left[h_{t-1},x_{t}\right]+b_{i}\right)\\ {\tilde{C}}_{t}=\tanh\left(W_{C}\cdot \left[h_{t-1},x_{t}\right]+b_{C}\right) \end{array}\right.$$

where $W_{i}$ and $W_{C}$ are the weight matrices of the sigmoid activation part and the tanh activation part in the input gate, and $b_{i}$ and $b_{C}$ represent the bias terms of the two activation parts. Combine the two parts:

(3) $$C_{t}=f_{t}*C_{t-1}+i_{t}*{\tilde{C}}_{t}$$

$C_{t}$ is the updated product of the combination of the current time and the long-term state ${\tilde{C}}_{t}$ and $C_{t-1}$ at the previous time. Then, the output $h_{t-1}$ at the previous time and $x_{t}$ at the current time are processed by the activation function sigmoid to obtain the output $o_{t}$:

(4) $$o_{t}=\sigma \left(W_{o}\cdot \left[h_{t-1},x_{t}\right]+b_{o}\right)$$

where $W_{o}$ is the weight matrix of the output gate, and $b_{o}$ is the bias matrix. Further, through the tanh activation function, the long-term state $C_{t}$ at the current moment is processed by the product of the value and $o_{t}$ to obtain the output $h_{t}$ at the current moment:

(5) $$h_{t}=o_{t}*\tanh\left(C_{t}\right)$$

Each one-dimensional input sample will obtain an output, and the sequential infrared signals corresponding to the L frame will obtain L output values $h_{t}$. The softmax [34] activation function is further used to normalize the output at the current moment:

(6) $${\hat{y}}_{t}=\tau \left(\sum Vh_{t}+c\right)$$

where ${\hat{y}}_{t}$ is the output value obtained after the above processing of the sequential infrared signals of the L frames, τ is the activation function softmax, *V* is the weight matrix and *c* is the bias matrix. Through the above formula, the output of each moment will be integrated into the calculation of the next moment. Thanks to the correlation between the input and output of the previous moment and the current moment in the LSTM, the network has the "memory" ability. Finally, the output ${\hat{y}}_{t}$ at the last moment is used as the final output. The ${\hat{y}}_{t}$ is a one-dimensional vector whose element values are between 0 and 1, and each element corresponds to an activity label. The element value is the probability value calculated by Formula (9). Next, compare the size of each element and output the element position *k* corresponding to the largest value. It is the activity tag value corresponding to the sequential infrared signal of the L frame. By comparing the output value with the label value, the gradient descent [35] method is used to iteratively update the parameters in the network through the backpropagation [36] process of the network. The collected sequential infrared signals will be trained through the above method. The model is tested and saved during each step of training. The training is over, and the optimal parameters and results obtained during the training process are fixed and saved.

### 2.5. Real-Time Detection

The collected infrared signals are sent to the server through the MQTT protocol in real time. Once the collected infrared signals reach the set number of frames, they are converted into a format that conforms to the model input and sent to the trained LSTM model to obtain probability values corresponding to the activities. The value of the tag corresponding to the largest probability is selected as the predicted value and input into the real-time detection module. It has a picture switching function in the real-time detection module. This function can switch to the corresponding picture according to the activity corresponding to the input value of the prediction and display the real-time activity through the picture. The received data are cleared and repeat the above steps to achieve real-time detection.

## 3. Experimental Evaluation

### 3.1. Experimental Setup and Data Description

**Figure 8:**
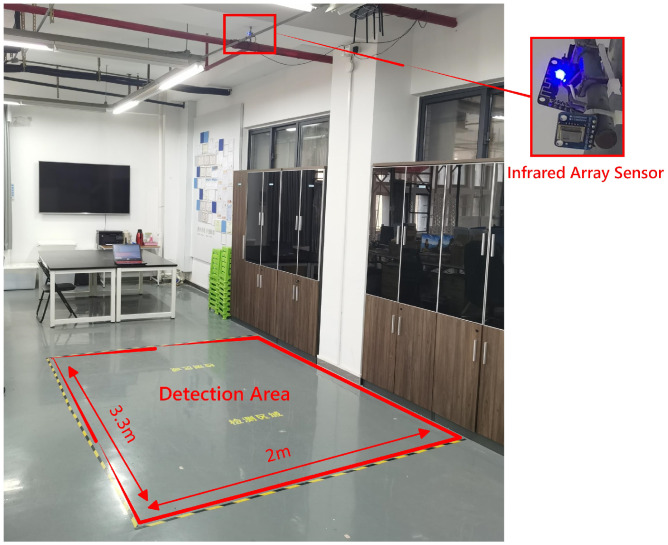
The layout of the experimental environment.

The proposed method was verified in an indoor environment, as shown in Figure 8. The detection area was in the rectangular red area in Figure 8, with an area of approximately 6.6 m${}^{2}$, and a length and width of 3.3 m and 2 m, respectively. The infrared array sensor of 8×8 pixels, Grid-EYE AMG8833, was installed about 3 m directly above the center of the detection area. The sensor was connected with ESP8266 for data collection, and the frequency was 20 frames/s. The infrared signal was trained and tested in the server, and it was uploaded to the server through a TP-Link886N router. The main hardware configuration of this server has the following: Intel Core i7-8750H CPU, 16G memory and an NVIDIA GeForce GTX1060 GPU. Regarding the software, the server was equipped with the Windows 10 operating system, the deep learning framework was based on Keras 2.2.4 and the construction of the network structure and programming were carried out in Python 3.6.4.

**Figure 9:**
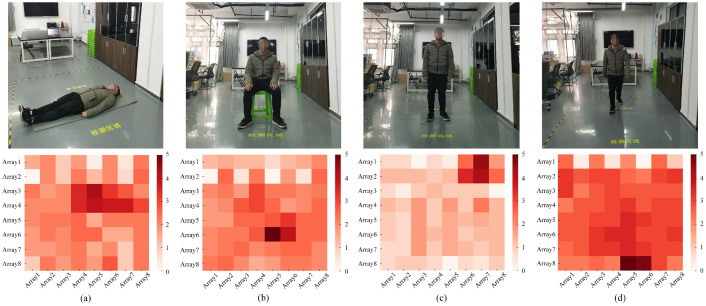
Four types of HAR and heatmaps corresponding to infrared signals. (**a**) Lying. (**b**) Sitting. (**c**) Standing. (**d**) Walking.

**Figure 10:**
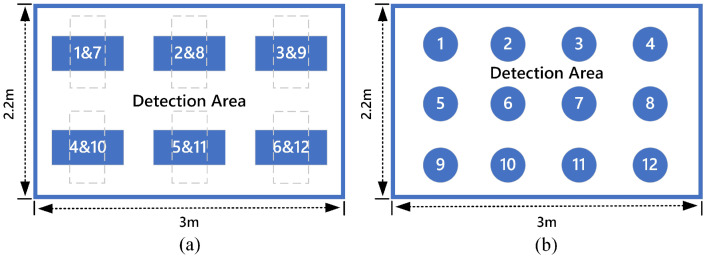
Schematic diagram of signal acquisition point. (**a**) Sampling point for lying. (**b**) Sampling point for sitting and standing.

There are five states of lying, standing, sitting, walking and empty. The four activities and the corresponding infrared signals’ heatmaps correspond to (a), (b), (c) and (d) in Figure 9. During the experiment, an experimenter acted as the experiment area. The experimenter was a male 175 cm tall and 75 kg in weight, a typical body shape in the population. The experimenter collected the infrared signals of lying at the six sampling points shown in Figure 10a. The infrared signals of standing and sitting were collected at 12 sampling points in Figure 10b. The human walked irregularly in the detection area to collect walking infrared signals. In the process of data acquisition, the participant performed different activities in as many different directions as possible. The dataset contains the signal in different directions for each activity.

The infrared signals with different activities in the room temperature range of 14 to $20{\;}^{\circ }C$ were fed into the model for training. The robustness and effectiveness of the model were improved. A low-pass Butterworth filter was utilized in this paper, and the cut-off frequency and order were 0.15 and 5, respectively. There were 43,200 frames of infrared signals collected in each of the five states, and the total of the infrared signals in the five states was 216,000 frames. Every 20 frames of infrared signals were taken as a sample, and the total number of samples was 10,800. After processing the collected data with the J-filter and the Butterworth filter mentioned in Section 2, the samples of each state were labeled. The label values of the five states of lying, standing, sitting, walking and empty correspond to 0, 1, 2, 3 and 4. The sample was randomly shuffled and divided into the training set and testing set according to the ratio of 80% and 20%.

### 3.2. Results

Before training, the sequential infrared signals need to be processed into a type suitable for model input. A sample has 20 frames of data, and each frame of data has 64 infrared signals. Each 20×64 dimension matrix signal is converted into a 1×1280 dimension vector. Therefore, there are 1280 nodes in the input layer of LSTM. Other structural parameters of LSTM are as follows: the number of nodes in the hidden layer is 640 and each hidden layer node is connected to a full connection layer with 100 backward nodes, and there is a ReLU [37] activation layer behind the full connection layer. Finally, the softmax activation function is used to normalize the data to obtain the output. Additionally, the timesteps of LSTM are set to 16.

**Figure 11:**
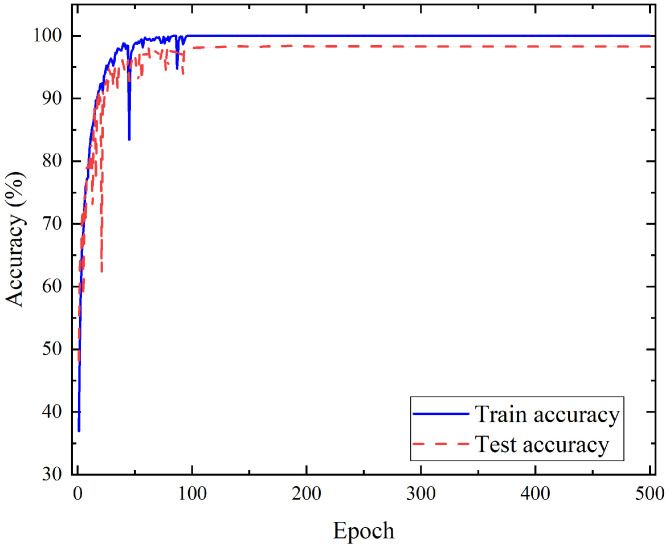
The results of each step in the model training process.

**Figure 12:**
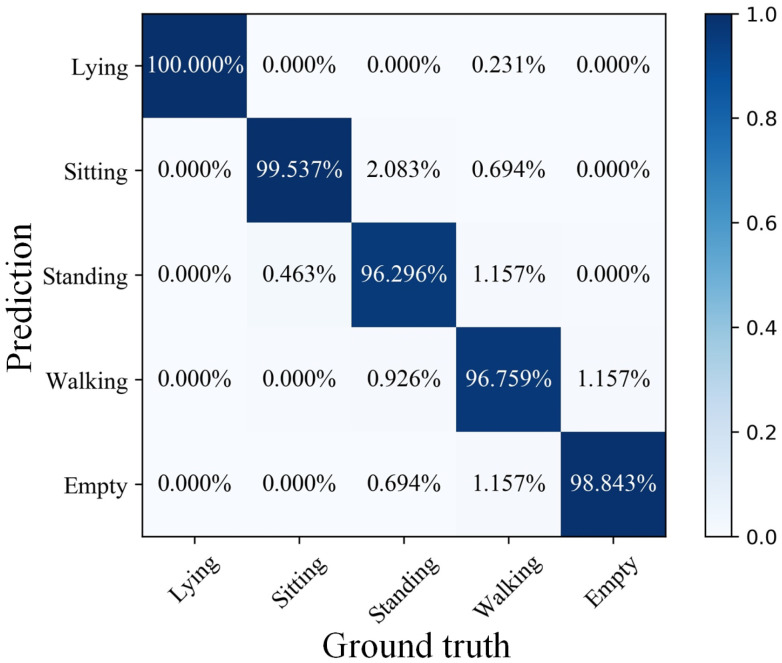
Confusion matrix of the final test result of the model.

The training steps total 500 times. After each training is completed, the testing set is used to test the effect of the model and save the training and test results of each step. The training and testing results of each step are plotted. As shown in Figure 11, the model was trained to step 99, and the recognition accuracy of the training set reached 100%. At the end of the training, the recognition accuracy of the testing set based on the model constructed by LSTM reached 98.287%. Specifically, it can be seen from the confusion matrix in Figure 12 that the test accuracy of the model for the five states is as high as 96% or more. In particular, the recognition accuracy of lying reached 100%, and only a very small number of false recognition cases occurred in the other states. Through the above results, the proposed method effectively extracts the data characteristics of infrared signals in different human activities and achieves high recognition accuracy for different human activities.

It is worth mentioning the tested samples include infrared signals collected at room temperature from 14 to $20{\;}^{\circ }C$. The effectiveness of the J-filter at different indoor temperatures is further verified. The strong robustness of the proposed method and model has been verified.

**Figure 13:**
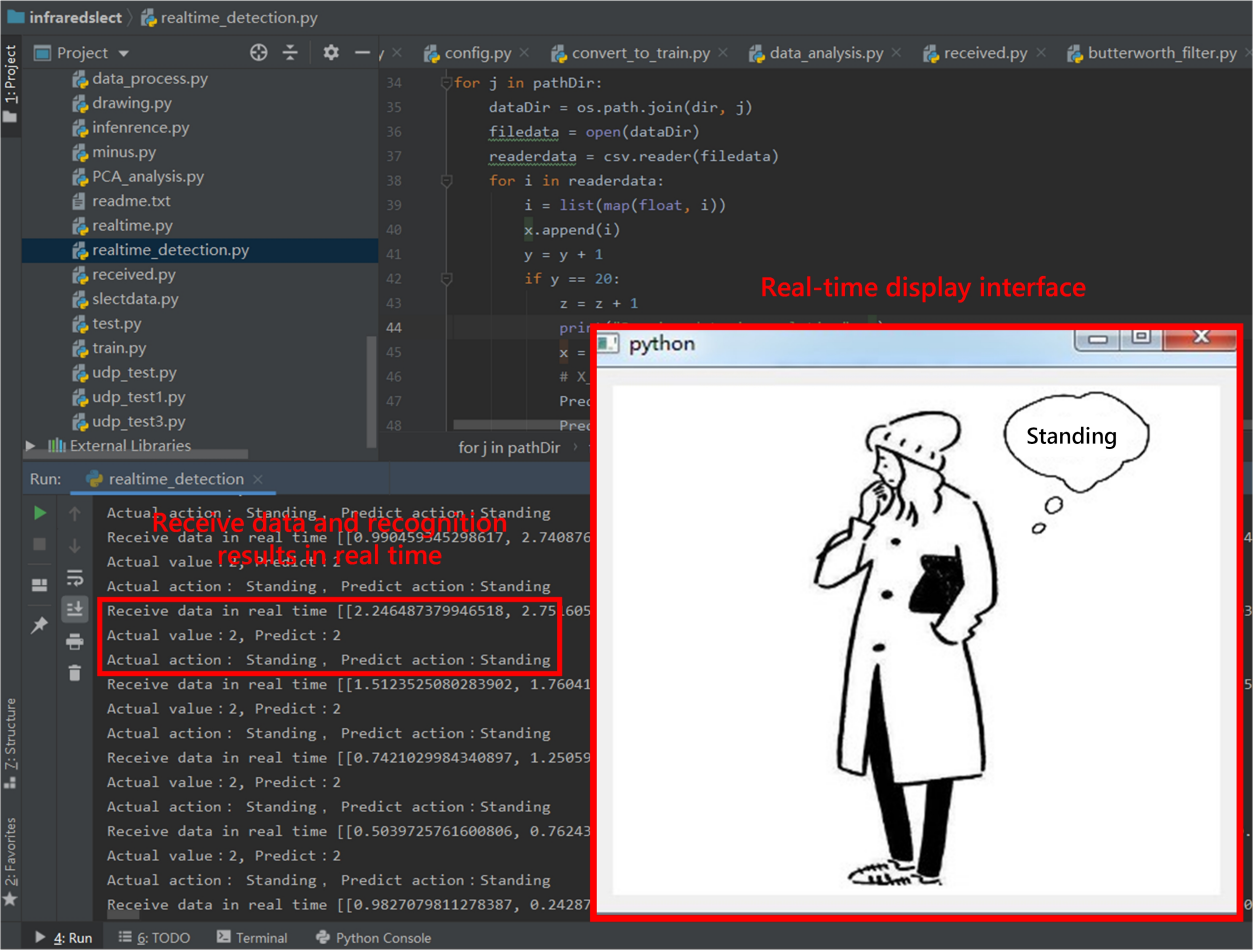
The interface of real-time detection program.

**Figure 14:**
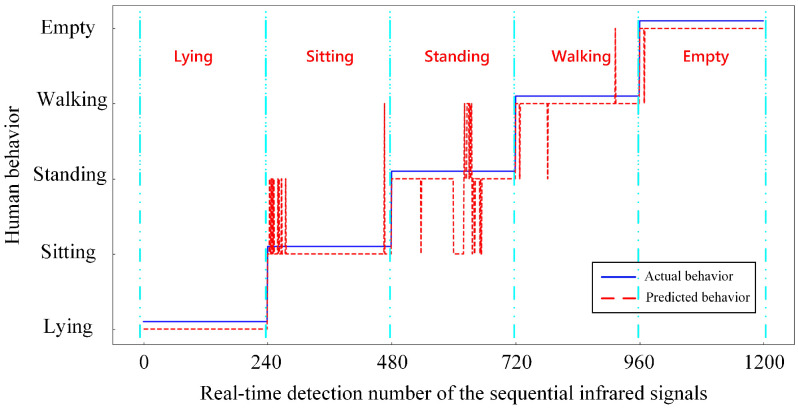
The real-time detection results corresponding to each activity.

The PyQt5 package was used to write a visualization program to verify the actual detection effect of the proposed method. PyQt5 uses Python to encapsulate all classes of Qt. The server obtains the infrared signals collected by the sensor by subscribing to MQTT. Once 20 frames of infrared signals are collected, they are processed by the J-filter and the Butterworth filter and sent to the trained model for detection. As shown in Figure 13, the real-time predicted value can be seen on the left, and the display interface on the right shows the picture corresponding to the recognized activity. Through the setting of the slot function, the picture switching function required for the compilation interface display is realized. The current activity of humans in the detection area is shown through the display interface. The real-time detection module is used to test the activity detection of an on-site human. During the real-time detection process, the experimenters performed the following four activities in the detection area: lying, standing, sitting and walking. The situation in the empty state is also detected. The experimental results are presented in Figure 14. The predicted value of real-time detection is consistent with the real movement, and it is very effective for real-time detection in lying, standing, walking and empty states. According to statistics, the real-time recognition accuracy rates of lying, standing, sitting, walking and empty states reached 100%, 96.250%, 85%, 98.750% and 99.583%, respectively, and the overall recognition accuracy reached 95.917%. The results show the proposed method is still effective in practical applications and achieves a high real-time detection accuracy. The high practical value of this method has been proved.

### 3.3. Impact of the Frames in the Sample

**Figure 15:**
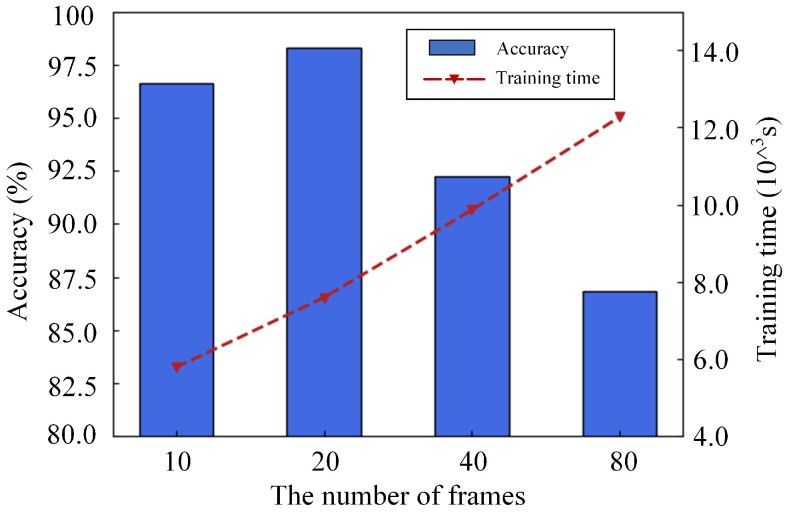
Experimental results of different numbers of frames.

The various frames of samples are used as research objects. Different numbers of frames are selected as samples for experimentation. The frame numbers of 10, 20, 40 and 80 are selected as the number of frames for each sample. The more frames the sample contains, the longer it takes to train the model, as shown in Figure 15. The increase in the number of frames does not lead to an increase in the accuracy of HAR but shows a downward trend when the number of frames exceeds 20. Ten frames of infrared signals are used as a sample, although the training time of the model is the shortest, and the 96.644% accuracy of the model at this time is not as good as the 98.287% accuracy of 20 frames as a sample. The experimental results are analyzed, and the sequential infrared signals’ characteristics corresponding to different human activities cannot be well extracted by the model with the length of signal segmentation being too small. Nonetheless, the cost of model training increases if the signal segmentation is too large. The number of frames for each sample of 20 is chosen after considering the model performance and training cost.

### 3.4. Impact of the Number of Hidden Layer Nodes

**Figure 16:**
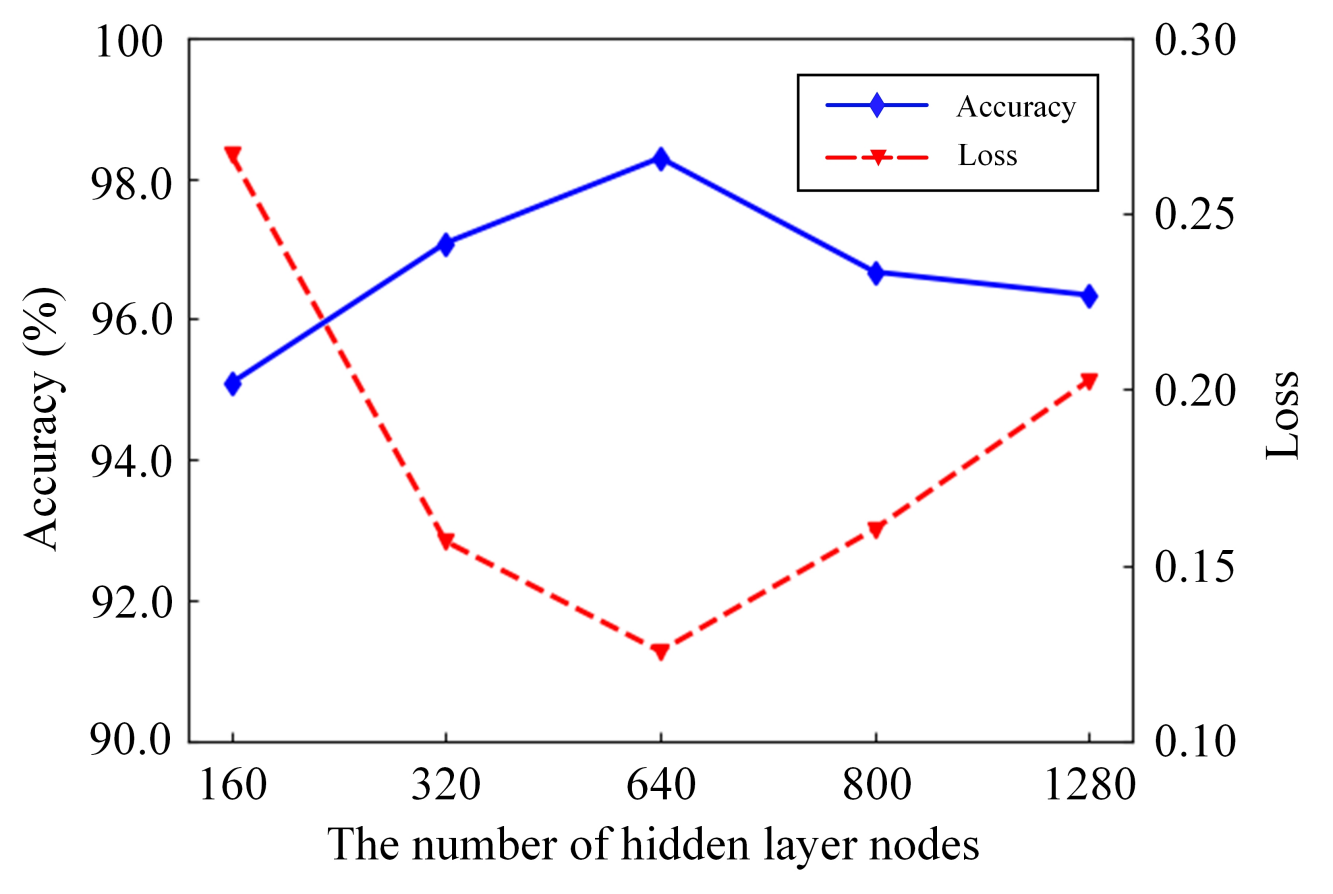
Experimental results of different numbers of hidden layer nodes.

Although some parameters of the deep learning network cannot play a decisive role in the effect of the training model, they still have a greater impact on the detection results. The impact of the number of hidden layer nodes on the model effect was researched. Five types of numbers are selected for the experiment, and the number of hidden layer nodes is set to 160, 320, 640, 800 and 1280. Figure 16 shows the experimental results under different numbers of hidden layer nodes. The number of hidden layer nodes increases from 160 to 640, and the test accuracy of the model increases from 95.093% to 98.287%. The loss of the model is also reduced from 0.2668 to 0.1257. The number of nodes is further increased, yet the test accuracy of the model shows a downward trend, and the loss value gradually increases. The increase in the number of hidden layer nodes improves the test accuracy and effectively extracts features of the data. The model also has better data-fitting capabilities and appears to over-fit, resulting in a decrease in test accuracy if there are too many nodes. The experimental results show the hidden layer nodes at 640 are the best choice.

### 3.5. Impact of the Timesteps

**Figure 17:**
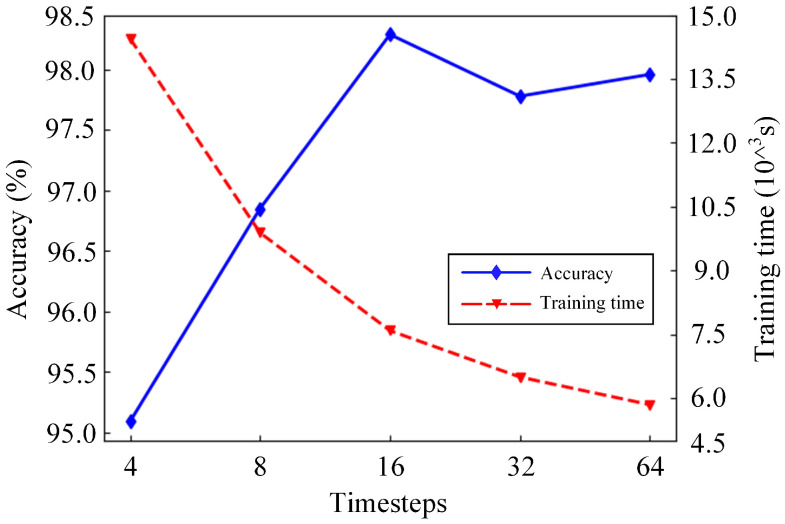
Experimental results of different timesteps.

Timesteps [38] are one of the important parameters of LSTM. The impact of timesteps on the model was studied. The timesteps were set to 4, 8, 16, 32 and 64 for model training. After 500 steps of training, the impact of the model on the accuracy of HAR under different timesteps can be seen in Figure 17. Although the recognition accuracy of the model was 95.046% with the timesteps set to 4, the training time of the model was the longest—the training model took $14.45\times 10^{\wedge 3}$ s. It is easy to find that the training time of the model decreases with the increase in timesteps, but the downward trend gradually slows down. However, the highest recognition accuracy reached 98.287%, and the training time was reduced to $7.59\times 10^{\wedge 3}$ s as the timesteps increased to 16. Compared with timesteps set to 4, the recognition accuracy of the model is not only improved by 3.241% but also the training time is reduced by nearly half. The recognition accuracy of the model did not increase with the increase in the timesteps but rather declined. The change in timesteps has an impact on the recognition accuracy of the model; the impact on the training time is particularly significant. Comprehensive consideration of the recognition accuracy and training time of the model and setting the timesteps to 16 represent a cost-effective choice.

### 3.6. Impact of the Filtering Algorithms

The impact of the preprocessing of infrared data on the experimental results is discussed. The effectiveness of the combination of the J-filter and the Butterworth filter method is also verified in this section. The raw data and the data processed by background subtraction, removing the average pixel temperature and just the J-filter were fed into the same model for training. The training results are shown in Table 1.

The data processed by the J-filter and the Butterworth filter have a higher accuracy, recall and F1-score than the others. Compared with background subtraction and the method of removing the average pixel temperature, the accuracy of the proposed method is improved by 7.731% and 6.018%, respectively. In addition, it can observed that the accuracy of just the J-filter reaches 96.644%, which is obviously better than background subtraction (90.556%) and removing the average pixel temperature (92.269%). It can be proved that the J-filter is a very suitable filtering approach for infrared signals.

### 3.7. Comparative Analysis

**Figure 18:**
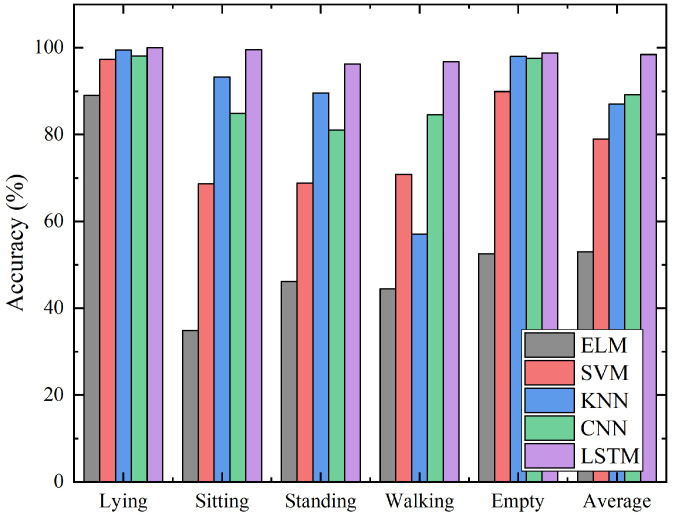
Comparison of the recognition results of various activities by different algorithms.

To further prove the efficiency of the proposed method, a comparison was carried out between the proposed approach based on LSTM and other machine learning methods. Extreme learning machine (ELM) [39], support vector machine (SVM) [40], k-nearest neighbors (KNN) [41] and convolutional neural networks (CNNs) [42] were selected to train and test the same sequential infrared signals. The recognition results of each algorithm in different activities are presented in Figure 18.

The recognition accuracy of the algorithms is ranked from high to low: for the recognition algorithms based on LSTM, CNN, KNN, SVM and ELM, the corresponding recognition accuracy is 98.287%, 89.242%, 87.490%, 79.108% and 53.402%. It is easy to observe the proposed method is higher than the other algorithms in terms of the recognition accuracy of a single activity and the overall recognition accuracy. The algorithm proposed can still achieve more than 96% accurate recognition of walking in the case of other algorithms misrecognizing walking. The equilibrium of the proposed algorithm to the recognition effect of different activities is verified. Compared with other algorithms, the advantages of the algorithm proposed are more obvious. The results in this paper are compared with wearable device-based [7], WiFi-based [17] and other infrared-based approaches [22,25,27]. The same activities, sitting and standing, are selected as the references and shown in Table 2. Compared with WiFi- and wearable device-based approaches, only one sensor is needed to achieve higher accuracy with the proposed method. The low-resolution infrared sensor proposed in this paper achieves accuracy similar to the higher-resolution infrared method. It can be observed that the proposed method can utilize low cost and, meanwhile, maintain the optimal performance.

## 4. Conclusions

This paper presented an HAR approach based on a low-resolution infrared array sensor using a deep learning network. In the proposed HAR method, the infrared thermal images of 8×8 are collected from the infrared sensor for recognition. In the data processing stage, the combination of the J-filter and the Butterworth filter is performed to reduce the noise of the raw infrared signal and eliminate the impact of temperature fluctuations. The infrared signal matrix is converted to a temporal sequence for the training stage. The LSTM model network is utilized to automatically extract the characteristics of the infrared signal and train the detection model. The results indicate that the daily activities of lying, standing, sitting, walking and empty can be accurately distinguished with an accuracy of 98.287%. Our approach yields a better performance compared with other machine learning methods. The proposed device-free HAR system offers a low-cost and high-accuracy sensing solution for application in privacy-preserving smart home scenarios. Future work is needed to extend the system to more participants and conduct the research about transition activities. The performance assessment of infrared sensors at different heights is also planned for the future.

## Figures and Tables

**Table 1:** Comparison of results with different data processing.

	Raw Data	Background Subtraction	Remove Average Temperature	J-Filter	J-Filter and Butterworh
Accuracy(%)	84.259	90.556	92.269	96.644	98.287
Recall(%)	84.080	90.498	92.226	96.658	98.290
F1-score(%)	84.169	90.527	92.247	96.651	98.289

**Table 2:** Comparison of the results between different resolutions.

Method	Number of Sensors	Pixel	Mean Accuracy
Accelerometer+SVM [7]	1	-	91.00%
CSI signals+HMM [17]	2	-	96.50%
PIR sensors+GS-HHMM [22]	4	8 × 16	91.00%
Thermal sensor+SRCNN [25]	1	120 × 160	92.31%
Thermal videos+CNN [27]	1	640 × 480	95.97%
The proposed method	1	8 × 8	97.92%

## Data Availability

Not applicable.

## References

[1] De-La-Hoz-Franco E., Ariza-Colpas P., Quero J.M., Espinilla M. (2018). Sensor-based datasets for Human Activity Recognition—A Systematic Review of Literature. IEEE Access.

[2] Zheng Y.L., Ding X.R., Poon C.C.Y., Lo B.P.L. (2014). Unobtrusive Sensing and Wearable Devices for Health Informatics. IEEE Trans. Biomed. Eng..

[3] De Backere O., Van den A., Nelis J., Bonte P., Clement E., Philpott M., Hoebeke J., Verstichel S., Ackaert A., De Turck F. (2015). Towards a social and context-aware multi-sensor fall detection and risk assessment platform. Comput. Biol. Med..

[4] Kern N., Antifakos S., Schiele B., Schwaninger A. A model for human interruptability: Experimental evaluation and automatic estimation from wearable sensors. Proceedings of the Eighth International Symposium on Wearable Computers.

[5] Khan A.M., Lee Y., Lee S.Y., Kim T. (2010). A Triaxial Accelerometer-Based Physical-Activity Recognition via Augmented-Signal Features and a Hierarchical Recognizer. IEEE Trans. Inf. Technol. Biomed..

[6] Wannenburg J., Malekian R. (2016). Physical Activity Recognition from Smartphone Accelerometer Data for User Context Awareness Sensing. IEEE Trans. Syst. Man Cybern. Syst..

[7] Bisio I., Delfino A., Lavagetto F., Sciarrone A. (2017). Enabling IoT for In-Home Rehabilitation: Accelerometer Signals Classification Methods for Activity and Movement Recognition. IEEE Internet Things J..

[8] Hu G., Qiu X., Meng L. RTagCare: Deep human activity recognition powered by passive computational RFID sensors. Proceedings of the 2016 18th Asia-Pacific Network Operations and Management Symposium (APNOMS).

[9] Hu G., Qiu X., Meng L. Human activity recognition based on Hidden Markov Models using computational RFID. Proceedings of the 2017 4th International Conference on Systems and Informatics (ICSAI).

[10] Wu Y. Research on bank intelligent video image processing and monitoring control system based on OpenCV. Proceedings of the 2009 3rd International Conference on Anti-Counterfeiting, Security, and Identification in Communication.

[11] Luo R.C., Wu X. Real-time Gender Recognition Based on 3D Human Body Shape for Human-Robot Interaction. Proceedings of the 2014 9th ACM/IEEE International Conference on Human-Robot Interaction (HRI).

[12] Ni B., Wang G., Moulin P. RGBD-HuDaAct: A color-depth video database for human daily activity recognition. Proceedings of the 2011 IEEE International Conference on Computer Vision Workshops (ICCV Workshops).

[13] Sanal Kumar K.P., Bhavani R. (2018). Human activity recognition in egocentric video using HOG, GiST and color features. Multimed. Tools Appl..

[14] Gupta A., Gupta K., Gupta K., Gupta K. A Survey on Human Activity Recognition and Classification. Proceedings of the 2020 International Conference on Communication and Signal Processing (ICCSP).

[15] Zhao K., Xi W., Jiang Z., Wang Z., Zhang X. Leveraging Topic Model for CSI Based Human Activity Recognition. Proceedings of the 2016 12th International Conference on Mobile Ad-Hoc and Sensor Networks (MSN).

[16] Damodaran N., Schäfer J. Device Free Human Activity Recognition using WiFi Channel State Information. Proceedings of the 2019 IEEE SmartWorld, Ubiquitous Intelligence Computing, Advanced Trusted Computing, Scalable Computing Communications, Cloud Big Data Computing, Internet of People and Smart City Innovation (SmartWorld/SCALCOM/UIC/ATC/CBDCom/IOP/SCI).

[17] Wang W., Liu A.X., Shahzad M., Ling K., Lu S. (2017). Device-Free Human Activity Recognition Using Commercial WiFi Devices. IEEE J. Sel. Areas Commun..

[18] Gonzalez L.I.L., Troost M., Amft O. (2013). Using a Thermopile Matrix Sensor to Recognize Energy-related Activities in Offices. Procedia Comput. Sci..

[19] Chen W.-H., Ma H.-P. A fall detection system based on infrared array sensors with tracking capability for the elderly at home. Proceedings of the 2015 17th International Conference on E-health Networking, Application Services (HealthCom).

[20] Yun J., Woo J. (2020). A Comparative Analysis of Deep Learning and Machine Learning on Detecting Movement Directions Using PIR Sensors. IEEE Internet Things J..

[21] Guan Q., Li C., Qin L., Wang G. (2019). Daily Activity Recognition Using Pyroelectric Infrared Sensors and Reference Structures. IEEE Sens. J..

[22] Guan Q., Yin X., Guo X., Wang G. (2016). A Novel Infrared Motion Sensing System for Compressive Classification of Physical Activity. IEEE Sens. J..

[23] Torres A. Adafruit AMG8833 8x8 Thermal Camera Sensor. https://cdn-learn.adafruit.com/downloads/pdf/adafruit-amg8833-8x8-thermal-camera-sensor.pdf.

[24] Gochoo M., Tan T.H., Batjargal T., Seredin O., Huang S.C. Device-Free Non-Privacy Invasive Indoor Human Posture Recognition Using Low-Resolution Infrared Sensor-Based Wireless Sensor Networks and DCNN. Proceedings of the 2018 IEEE International Conference on Systems, Man, and Cybernetics (SMC).

[25] Shih C.S., Wang Y.T., Chou J.J. Multiple-Image Super-Resolution for Networked Extremely Low-Resolution Thermal Sensor Array. Proceedings of the 2020 IEEE Second Workshop on Machine Learning on Edge in Sensor Systems (SenSys-ML).

[26] Uddin M.Z., Torresen J. A Deep Learning-Based Human Activity Recognition in Darkness. Proceedings of the 2018 Colour and Visual Computing Symposium (CVCS).

[27] Batchuluun G., Nguyen D.T., Pham T.D., Park C., Park K.R. (2019). Action Recognition from Thermal Videos. IEEE Access.

[28] Panasonic (2017). Infrared Array Sensor Grid-EYE. https://cdn.sparkfun.com/assets/4/1/c/0/1/Grid-EYE_Datasheet.pdf.

[29] Kodali R.K., Mahesh K.S. A low cost implementation of MQTT using ESP8266. Proceedings of the 2016 2nd International Conference on Contemporary Computing and Informatics (IC3I).

[30] Ali A.S., Radwan A.G., Soliman A.M. (2013). Fractional Order Butterworth Filter: Active and Passive Realizations. IEEE J. Emerg. Sel. Top. Circuits Syst..

[31] Hochreiter S., Schmidhuber J. (1997). Long Short-Term Memory. Neural Comput..

[32] Koutník J., Greff K., Gomez F., Schmidhuber J. (2014). A Clockwork RNN. Comput. Sci..

[33] Godfrey L.B., Gashler M.S. Neural decomposition of time-series data. Proceedings of the 2017 IEEE International Conference on Systems, Man and Cybernetics (SMC).

[34] Mohammed A.A., Umaashankar V. Effectiveness of Hierarchical Softmax in Large Scale Classification Tasks. Proceedings of the 2018 International Conference on Advances in Computing, Communications and Informatics (ICACCI).

[35] Lu Y., Salem F.M. Simplified gating in long short-term memory (LSTM) recurrent neural networks. Proceedings of the 2017 IEEE 60th International Midwest Symposium on Circuits and Systems (MWSCAS).

[36] Zhang J.R., Zhang J., Lok T.M., Lyu M.R. (2007). A hybrid particle swarm optimization–back-propagation algorithm for feedforward neural network training. Appl. Math. Comput..

[37] Clevert D.A., Unterthiner T., Hochreiter S. (2015). Fast and Accurate Deep Network Learning by Exponential Linear Units (ELUs). arXiv.

[38] Monner D., Reggia J.A. (2012). A generalized LSTM-like training algorithm for second-order recurrent neural networks. Neural Netw..

[39] Siew Z.C.K. (2006). Extreme learning machine: Theory and applications. Neurocomputing.

[40] Zhan Y., Shen D. (2005). Design efficient support vector machine for fast classification. Pattern Recognit.

[41] Kramer O. (2013). K-Nearest Neighbors. Intell. Syst. Ref. Libr..

[42] Arandjelovic R., Gronat P., Torii A., Pajdla T., Sivic J. (2017). NetVLAD: CNN architecture for weakly supervised place recognition. IEEE Trans. Pattern Anal. Mach. Intell..

